# Simulation and Control of the Formation of Ethyl Carbamate during the Fermentation and Distillation Processes of Chinese Baijiu

**DOI:** 10.3390/foods12040821

**Published:** 2023-02-15

**Authors:** Yuhang Di, Jianghua Li, Jian Chen, Xinrui Zhao, Guocheng Du

**Affiliations:** 1 Science Center for Future Foods, Jiangnan University, 1800 Lihu Road, Wuxi 214122, China; 2 Key Laboratory of Industrial Biotechnology, Ministry of Education, School of Biotechnology, Jiangnan University, 1800 Lihu Road, Wuxi 214122, China; 3 Jiangsu Province Engineering Research Center of Food Synthetic Biotechnology, Jiangnan University, 1800 Lihu Road, Wuxi 214122, China; 4 Engineering Research Center of Ministry of Education on Food Synthetic Biotechnology, Jiangnan University, 1800 Lihu Road, Wuxi 214122, China; 5 Key Laboratory of Carbohydrate Chemistry and Biotechnology, Ministry of Education, Jiangnan University, 1800 Lihu Road, Wuxi 214122, China

**Keywords:** Baijiu, ethyl carbamate, precursor, urea, cyanide, simulation, fermentation, distillation

## Abstract

Baijiu is a popular alcoholic beverage with a long history in China. However, the widespread presence of the ethyl carbamate (EC) carcinogen has raised many food safety concerns. To date, the main precursors of EC and its formation process have not been determined, resulting in difficulty controlling EC in Baijiu. In this study, the main precursors of EC are identified as urea and cyanide during the process of brewing for different flavors of Baijiu, while the dominant stage in which EC formation occurs is during the process of distillation rather than fermentation. In addition, the effects of temperature, pH value, alcohol concentration and metal ions on the formation of EC are confirmed. In the following study, the main precursor of EC is identified as cyanide during the process of distillation, and a combination of optimizing the distillation device and adding copper wire is proposed. Furthermore, the effect of this novel strategy is examined in gaseous reactions between cyanide and ethanol, reducing the concentration of EC by 74.0%. Finally, the feasibility of this strategy is verified in simulated distillations of fermented grains, reducing the formation of EC by 33.7–50.2%. This strategy has great application potential in industrial production.

## 1. Introduction

Chinese Baijiu is one of the oldest distilled spirits in the world and has been passed down for thousands of years [1]. The output and sale of Chinese Baijiu reached 7.16 million kiloliters and USD 847.1 billion in 2021 (China Industry Information Network, 2021). Thus, manufacturers are paying increasing attention to further improving the quality of Chinese Baijiu to meet the increasing demands for food safety and flavor variety. Among the thousands of compounds in Chinese Baijiu, ethyl carbamate (EC) is a class 2A carcinogen that is widely found in different types of Baijiu (including sauce flavor, strong flavor and light flavor) [2] and the level of EC can reach 382.33 μg/L [3], seriously threatening the health of consumers. As the limit of EC in distilled spirits is 150 μg/L in Canada, America and other European [4], it is necessary to control the content of EC in Baijiu by deeply clarifying the mechanism of EC formation.

It is known that cyanide and urea are the two main precursors of EC in Baijiu [5], but details of the formation pattern for these two precursors during the production of Baijiu are not clear. In previous research on other alcoholic beverages, there have been three common pathways to the formation of EC from urea. Firstly, EC can be formed at ambient temperature through the mild and spontaneous reaction between urea and ethanol (Equation (1)) [6,7]. In addition, a portion of urea can be decomposed into cyanate and isocyanate at 60–100 °C; then, cyanate and isocyanate can react with ethanol to form EC under acidic conditions (Equation (2)). Furthermore, another portion of urea can be decomposed into ammonia and cyanic acid at high temperatures. Additionally, gaseous cyanic acid can react with evaporated ethanol to form EC (Equation (3)) [7,8]. As for the other precursor, there are two common pathways to the formation of EC from cyanide. First, cyanide is catalyzed by copper ions to produce isocyanate; then, EC is formed through the reaction between isocyanate and ethanol, as shown in Equation (4) [9]. Additionally, cyanide can release gaseous HCN at high temperatures, and HCN is oxidized to isocyanic acid, which can react with gaseous ethanol to form EC (Equation (5)) [8,10]. However, due to the unique manufacturing techniques and diverse flavor features in Baijiu production, the main precursors of EC and its dominant forming process are complicated and still unclear.

(1) $${\mathrm{NH}}_{2}{\mathrm{CONH}}_{2}{+C}_{2}H_{5}{\mathrm{OH}=H}_{2}{\mathrm{NCO}}_{2}C_{2}H_{5}{+\mathrm{NH}}_{3}\;$$

(2) $${\mathrm{CNO}}^{-}{+C}_{2}H_{5}{\mathrm{OH}+H}^{+}{=H}_{2}{\mathrm{NCO}}_{2}C_{2}H_{5}\;$$

(3) $${\mathrm{NH}}_{2}{\mathrm{CONH}}_{2}{=\mathrm{NH}}_{3}+\mathrm{HCNO}\;$$

(4a) $${4\mathrm{CN}}^{-}+2\mathrm{Cu}(\mathrm{II}{)=2\mathrm{Cu}(\mathrm{CN})}_{2}\;$$

(4b) $${2\mathrm{Cu}(\mathrm{CN})}_{2}{=2\mathrm{Cu}(\mathrm{CN})+C}_{2}N_{2}\;$$

(4c) $$C_{2}N_{2}{+2\mathrm{OH}}^{-}{=\mathrm{OCN}}^{-}{+\mathrm{CN}}^{-}{+H}_{2}O\;$$

(4d) $${\mathrm{OCN}}^{-}{+C}_{2}H_{5}{\mathrm{OH}+H}^{+}{=H}_{2}{\mathrm{NCO}}_{2}C_{2}H_{5}\;$$

(5) $$\mathrm{HCN}+\frac{1}{2}O_{2}=\mathrm{HOCN}\;$$

In addition to the mechanism of EC formation, researchers have placed emphasis on approaches to reduce the content of EC in alcoholic beverages, including the refinement of raw materials, the screening of microorganisms that can degrade EC or its precursors, metabolic engineering of the brewing strains, and the optimization of manufacturing processes, etc. However, there is no effective method to remove precursors from raw materials, and the effect of pretreating raw materials on the nutritional value and fermentation process should be determined [11]. In addition, mixed cultures of several special microorganisms have been applied to reduce EC and its precursors, including *Bacillus amyloliquefaciens* J10 and *Wickerhamomyces anomalus* M11, which were screened from a distillery in the Sichuan province of China [12]; *Lysinibacillus sphaericus* MT33 screened from a distillery in Shandong province of China [13]; and *Saccharomyces cerevisiae* MT-1 [14] and JZ109 [15] obtained from the Chinese microbiological culture collection center. However, the introduction of new microorganisms to the stable microbial system may negatively affect the quality and flavor of Baijiu. Moreover, the overexpression of the *DUR3* gene in an industrial yeast strain using the CRISPR/Cas9 method [16] and the blocking of the synthetic pathway of urea via the sequential deletion of two *CAR1* alleles in a WY1 diploid wine yeast [17] can be used to reduce the content of EC; however, these approaches bring serious risks to food safety. Currently, an effective control strategy for EC in spirits is repeated distillation. For example, 94–98.5% EC can be removed through double-distillation in the production of sugarcane spirits [18]; however, repeated distillation will lead to great losses in both yield and flavor. Therefore, it is necessary to find more reasonable and effective strategies to control the content of EC in Baijiu.

Previous studies have shown that the primary metabolic pathways for EC formation in Baijiu are the urea–ethanol and cyanide–ethanol pathways [19]. However, studies on the mechanism of EC formation from precursors during production are limited, and there are few reports related to improving distillation processes to reduce EC content in Baijiu. Thus, in this study, the process of fermentation and distillation were simulated and the contents of EC and its precursors were detected to identify the real mechanism of EC formation in the three most important types of Baijiu (sauce flavor, strong flavor and light flavor) [20]. Based on the results, the new strategies applied during the processes of fermentation and distillation were effective in controlling the content of EC in Baijiu.

## 2. Materials and Methods

### 2.1. Materials and Chemicals

Special *Daqu*, which contain various microorganisms and enzymes that expedite the processes of saccharification and fermentation, were provided by the manufacturers of sauce-, strong- and light-flavor Baijiu. The dry yeast for brewing was purchased from Angel Yeast Co., Ltd. (Yichang, China). The standard substances for the analysis of cyanide in water were purchased from the Northern Wei Ye Institute of Metrology and Technology (Beijing, China). Urea, ethanol, hydrochloric acid, sodium acetate, acetonitrile, phosphoric acid, sodium hydroxide, dichloromethane and methanol were purchased from the Sinopharm Chemical Reagent Co. (Shanghai, China). Chloramine-T and 9-Hydroxyxanthene were produced by the McLean Reagent Co. (Shanghai, China). All solvents and reagents were analytical grade.

### 2.2. The Simulation of Baijiu Fermentation

To simulate the solid–state fermentation of Baijiu, three stages were performed sequentially: grain steaming, mixing of the liquor malt and fermentation. For the fermentation of sauce-, strong- and light-flavor Baijiu, 84 g of intact sorghum, 36 g of smashed sorghum, 72 g of water and 7.2 g of rice husk were added together to a 250 mL conical flask. The mixed materials were put into a sterilizing pot to steam the grains for 1 h, and then 2.5 mL of activated dry yeast powder and 10 g *Daqu* of the sauce flavor, strong flavor and light flavor were added, respectively. The final samples were sealed in conical flasks and transferred to an incubator at 37 °C for 30 days.

### 2.3. The Simulation of Aqueous Reactions between Urea and Ethanol or Cyanide and Ethanol during Distillation

#### 2.3.1. The Effect of Temperature on the Formation of EC and on Urea and Cyanide Residue

Different concentrations of urea (5, 10 and 20 mg/L) or cyanide (1, 2 and 4 mg/L) were prepared in an ethanol solution (2%) at pH 6.0 and reacted in a water bath at 80 °C, 90 °C and 100 °C, respectively. Samples were taken at 30 min, 60 min and 90 min for each reaction.

#### 2.3.2. The Effect of pH Value on the Formation of EC and on Urea and Cyanide Residue

Concentrations of 5 mg/L urea or 1 mg/L cyanide were prepared in ethanol solution (2%) at different pH values (3.0, 4.0, 5.0, 6.0, 7.0, 8.0 and 9.0) and reacted in a water bath at 100 °C. Samples were taken at 90 min for each reaction.

#### 2.3.3. The Effect of Ethanol Concentration on the Formation of EC and on Urea and Cyanide Residue

Concentrations of 5 mg/L urea or 1 mg/L cyanide were prepared in solutions with different concentrations of ethanol (2%, 4%, 6%, 8% and 10%) at a pH of 6.0 and reacted in a water bath at 100 °C. Samples were taken at 90 min for each reaction.

#### 2.3.4. The Effect of Metal Ions on the Formation of EC and on Urea and Cyanide Residue

Concentrations of 5 mg/L urea or 1 mg/L cyanide with 5 mmol/L different metal ions (Cu^2+^, Mn^2+^, Zn^2+^, Ca^2+^, Fe^2+^ and Mg^2+^) were prepared in an ethanol solution (2%) at a pH of 6.0 and reacted in a water bath at 100 °C. Samples were taken at 90 min for each reaction.

### 2.4. The Simulation of Gaseous Reactions between Urea and Ethanol or Cyanide and Ethanol during Distillation

#### 2.4.1. The Effect of Different Concentrations of Urea and Cyanide on the Formation of EC

**Figure 1:**
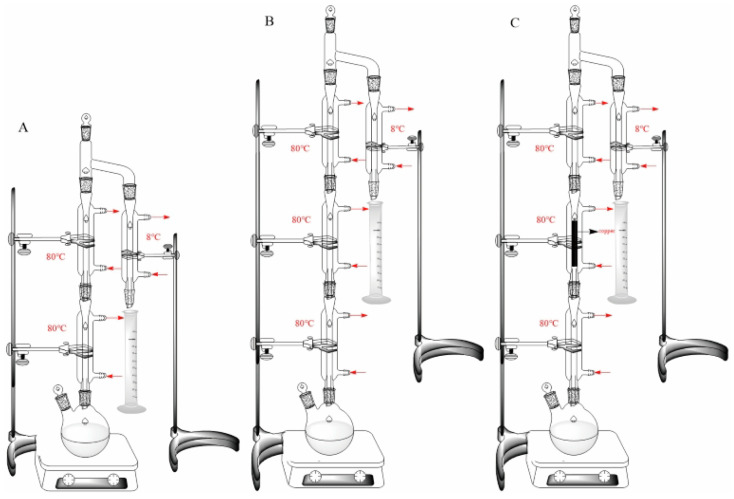
(**A**) The simulated device for distillation; (**B**) the optimized simulated device with an additional condenser tube for distillation; (**C**) the optimized simulated device with the addition of copper wire for distillation.

Different concentrations of urea (0.0001, 0.01, 0.05, 0.1, 0.5, 1.0, 5.0, 10.0, 15.0 and 20.0 mg/L) or cyanide (0.0001, 0.01, 0.05, 0.1, 0.5, 1.0, 2.0 and 4.0 mg/L) with 5 mmol/L Cu^2+^ were prepared in an ethanol solution (20%) at a pH of 3.0 in round-bottom flasks. The distillation for each sample was carried out by heating them in the simulated device (Figure 1A), and the distillate was collected after 10 min for use in the assay.

#### 2.4.2. The Effect of the Optimized Distillation Device and Copper Wires on the Formation of EC

Different concentrations of urea (1.0, 5.0 and 15.0 mg/L) or cyanide (1.0, 2.0 and 4.0 mg/L) with 5 mmol/L Cu^2+^ were prepared in an ethanol solution (20%) at a pH of 3.0 in round-bottom flasks. The distillation for each sample was carried out by heating them in the optimized simulated devices (Figure 1B,C), and the distillate was collected after 10 min for the detection of EC.

### 2.5. The Simulation of Solid Distillation for Baijiu

A total of 100 g of fermented grains was obtained at the end of the simulated fermentation of light-, strong- and sauce-flavor Baijiu and was put into a distiller that was connected to the distilling apparatus shown in Figure 1A,C, instead of into the round-bottom flask for heating and distillation. The distilled spirits were collected after 10 min for the detection of EC.

### 2.6. Analytical Method

Different methods were selected for pretreatment according to the status of the samples. For the solid fermented grains, 10 g samples were mixed with 10 mL of ultrapure water and were homogenized in an ultrasonic shaker for 15 min. Then, the samples were centrifuged at 8000 r/min for 10 min, and the supernatant was collected for the detection of EC and its precursors. As for the aqueous samples, they were filtrated and used for the following analysis.

To detect the concentration of urea, 0.4 mL of the sample, 0.6 mL of 9-Hydroxyxanthene and 0.1 mL of 0.1 mol/L hydrochloric acid were mixed well and reacted for 30 min in a centrifuge tube. Then, the reaction mixture was filtered through a 0.22 μm organic membrane and detected using HPLC-FID (Thermo, Shanghai, China) with a C18 column (4.6 mm × 150 mm). The sample was detected at an excitation wavelength of 213 nm and an emission wavelength of 308 nm, and the mobile phase was prepared according to the previous method [21]. To detect the concentration of cyanide, high-resolution gas chromatography–mass spectrometry (GC-MS, Thermo, Shanghai, China) was applied with a wax column (30 m × 0.25 mm × 0.25 μm), and the headspace injection method was used according to the national standard (GB 5009.36-2016). To detect the concentration of EC, a 2 mL sample was loaded into a solid-phase extraction column (alkaline diatomaceous earth) for 10 min, and then eluted using 15 mL of dichloromethane. The eluent was collected in a clean tube and dried using a rotary evaporator at room temperature. The solid residue was dissolved in 1 mL of methanol for analysis via ISQ GC-MS (Thermo, Shanghai, China) with a wax column (60 m × 0.25 mm × 0.25 μm) [22].

## 3. Results and Discussion

### 3.1. The Concentrations of EC, Urea and Cyanide during the Simulated Fermentation of Different Flavors of Baijiu

**Figure 2:**
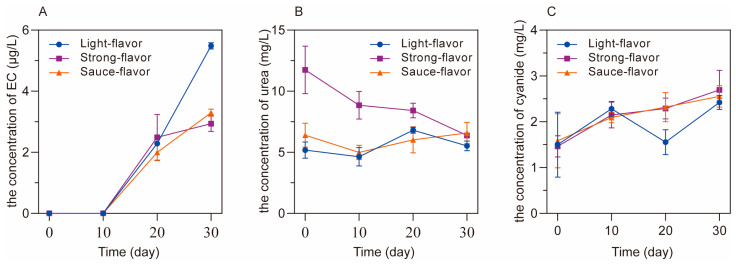
The concentration of EC (**A**), urea (**B**) and cyanide (**C**) during the simulated solid-state fermentation of light- (●), strong- (■) and sauce-flavor (▲) Baijiu.

To investigate the forming process of EC during the production of Baijiu, the fermentations of light-, strong- and sauce-flavor Baijiu were first simulated, and the concentration of EC and its precursors were monitored every 10 days during the process of fermentation. The levels of EC and its precursors are shown in Figure 2.

During the simulated fermentation, the concentration of EC increased in all three flavors of Baijiu, and the formation of EC mainly occurred in the middle and late stages of fermentation. The final concentration of EC remained in the range of 2–6 μg/L at the end of fermentation. As for the precursors of EC, the concentrations of urea (4–14 mg/kg) and cyanide (0.5–3.0 mg/kg) remained nearly stable during fermentation. According to Equations (1) and (4), urea and cyanide in the simulated system could theoretically generate 5.9–20.8 mg/kg and 0.4–2.6 mg/kg of EC, which was significantly higher than the actual quantity of EC. The reason for this phenomenon is that ethanol, as another precursor of EC, was formed in the middle and late stages of fermentation. In addition, during the process of simulated fermentation, urea slowly reacted with ethanol to form EC, as shown in Equation (1), under the lower temperature (37 °C). Moreover, the acidic condition in the simulated system was not suitable for the formation of EC through the reaction between cyanide and ethanol at 37 °C (Equation (4), requiring an alkaline condition and a copper catalyst. Furthermore, as the boiling point of EC is 182 °C, it is difficult for the lower concentration of EC formed in the fermented grains to be brought into the final distilled Baijiu. Therefore, the process of fermentation is not the main stage of EC formation, and the higher concentration of EC in Baijiu may be formed in the following process of distillation [3].

### 3.2. The Effects of Temperature, pH, Ethanol and Metal Ions on the Formation of EC during the Aqueous Reaction between Precursors and Ethanol

#### 3.2.1. The Concentrations of EC and Urea after Aqueous Reactions at Different Temperatures

**Figure 3:**
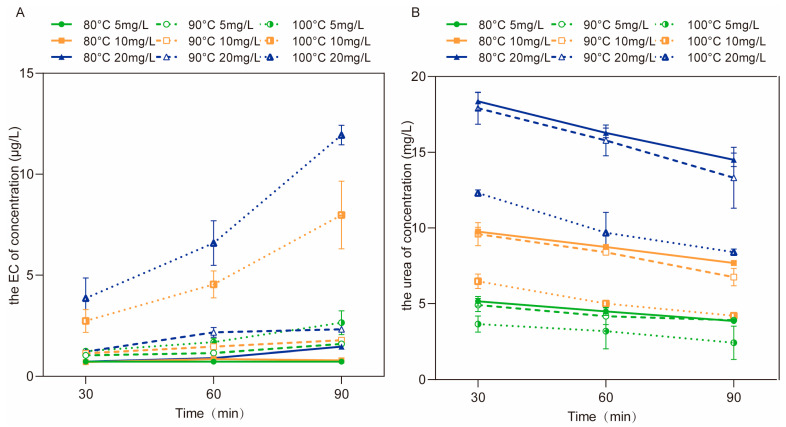
The concentrations of EC (**A**) and urea (**B**) during the aqueous reactions between urea (5.0, 10.0 and 20.0 mg/L) and ethanol (2%) at 80 °C, 90 °C and 100 °C, respectively.

The previous results showed that urea could react with ethanol to form EC (Equations (1) and (2)), and the concentration of urea was negatively correlated with the reaction time, while the amount of EC was positively correlated with the reaction time [23]. During the process of the simulated aqueous reactions in this study, the concentration of urea gradually decreased, and the concentration of EC gradually increased. Regarding the effect of the reaction temperature on the formation of EC, there was little significance in the difference between the reduction in urea at 80 °C and 90 °C, and the concentration of EC was lower than 2.5 μg/L, even after 90 min of reaction (Figure 3A). In addition, the reaction rate between urea and ethanol was rather slow (Figure 3B). However, when the reaction temperature reached 100 °C, the rate of EC formation increased significantly, and the final concentration of EC exceeded 11 μg/L in 90 min (Figure 3A). On the other hand, the reduction in urea was increased (Figure 3B). Moreover, along with the increasing concentration of urea, the reaction became violent, and the formation of EC was accelerated, which has also been previously verified [24]. Therefore, the formation of EC can be reduced during Baijiu fermentation by controlling the temperature and the concentration of urea.

#### 3.2.2. The Effects of pH Value, Alcohol Concentration and Metal Ions on the Formation of EC and the Residue of Urea

**Figure 4:**
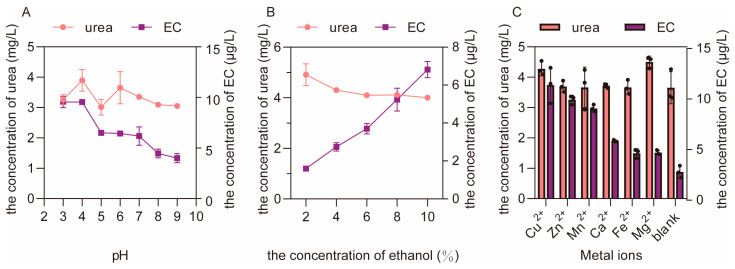
The effects of pH value (**A**), alcohol concentration (**B**) and metal ions (**C**) on the formation of EC and the residue of urea in aqueous reactions.

Compared with temperature, pH value had a more obvious effect on the formation of EC, and the concentration of EC increased when the pH value decreased (Figure 4A), indicating that the acidic condition was more suitable for the formation of EC. In addition, a higher concentration of ethanol can accelerate the reaction between urea and ethanol, leading to a decrease of urea residue and a significant increase in the formation of EC (Figure 4B). This suggests that a higher concentration of ethanol in the fermented grain will promote the reaction between urea and ethanol to form EC, which is consistent with previous research [25]. In addition to pH values and alcohol concentrations, all the metal ions (Cu^2+^, Mn^2+^, Zn^2+^, Ca^2+^, Fe^2+^ and Mg^2+^) tested in this study had positive effects on the formation of EC, among which the highest amount of EC was formed under the catalysis of Cu^2+^ (Figure 4C). Generally, in the real process of Baijiu fermentation, the continuous accumulation of EC in fermented grains can be reduced by controlling these essential influencing factors (pH value and the concentrations of ethanol and Cu^2+^ in fermented grains).

#### 3.2.3. The Concentrations of EC and Cyanide after Aqueous Reactions at Different Temperatures

**Figure 5:**
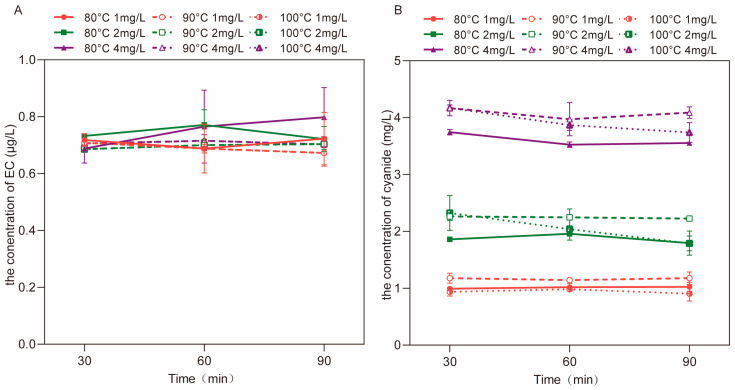
The concentrations of EC (**A**) and cyanide (**B**) during aqueous reactions between cyanide (1.0, 2.0 and 4.0 mg/L) and ethanol (2%) at 80 °C, 90 °C and 100 °C, respectively.

The results show that temperature and the concentration of cyanide had no noticeable effect on the formation of EC (Figure 5A), and cyanide reduction was extremely slow (Figure 5B), even after the reaction at 100 °C for 90 min. The highest concentration of EC could only reach 0.92 μg/L (Figure 5A), indicating that it is inefficient to form EC through a direct reaction between cyanide and ethanol under aqueous conditions.

#### 3.2.4. The Effects of pH Value, Alcohol Concentration and Metal Ions on the Formation of EC and the Residue of Cyanide

**Figure 6:**
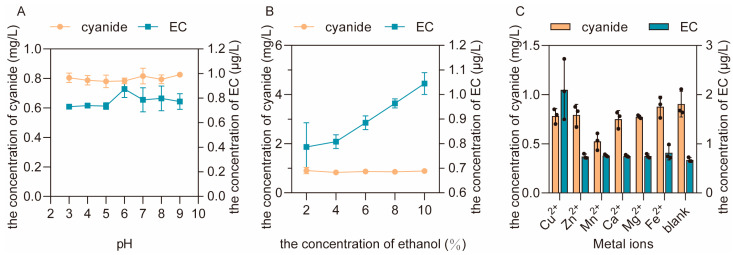
The effects of pH value (**A**), alcohol concentration (**B**) and metal ions (**C**) on the formation of EC and the residue of cyanide in aqueous reactions.

The results show that different pH values had no effect on the formation of EC (Figure 6A), but a higher concentration of ethanol can promote the reaction between cyanide and ethanol (Figure 6B). It is worth noting that only Cu^2+^ had a significant positive effect on the formation of EC among all the tested metal ions (Figure 6C), further proving the catalytic role of Cu^2+^ in the reaction between cyanide and ethanol [26]. Thus, the concentration of EC in the fermented grains can also be reduced by controlling the concentrations of Cu^2+^ and ethanol in the real process of Baijiu fermentation.

### 3.3. The Effect of Different Precursors and Devices on the Formation of EC during Gaseous Reactions

#### 3.3.1. The Concentration of EC at Different Initial Concentrations of Urea and Cyanide during Distillation

**Figure 7:**
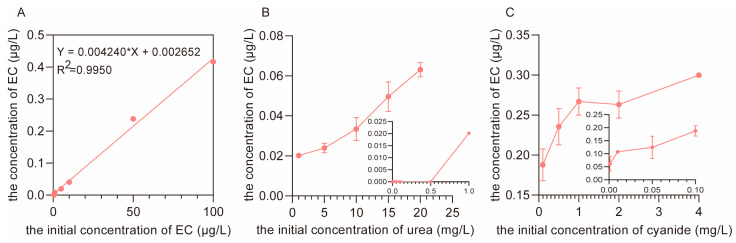
The concentration of EC in the distillate after 10 min of distillation at 100 °C. (**A**) Co-distillation of ethanol with different concentrations of EC. (**B**) Co-distillation of ethanol with different concentrations of urea. (**C**) Co-distillation of ethanol with different concentrations of cyanide.

As shown in Figure 7A, only a small portion of EC (lower than 0.5%) could be transferred into the distillate in the simulated process of distillation [27]. Thus, the EC in Baijiu was predominantly formed from the gaseous derivates of urea and cyanide in the distillation stage at a high temperature. Compared with the concentration of EC (0.024 μg/L) formed from urea (5.0 mg/L in the fermented grains), cyanide (2.0 mg/L in the fermented grains) formed nearly 10 times more EC (0.263 μg/L) after 10 min of reaction with ethanol at 100 °C (Figure 7B,C) and can be considered the main precursor of EC during the process of distillation. In addition, the concentration of EC increased along with increasing concentrations of urea and cyanide in the simulated system of distillation but became stable when the concentration of cyanide exceeded 1.0 mg/L (Figure 7B,C). Hence, controlling the concentrations of cyanide or urea in fermented grains is a common strategy to eliminate EC in Baijiu [11].

The main reason for the faster reaction rate of cyanide with ethanol in the gaseous simulated system is that urea can be decomposed into HCNO, while cyanide can be oxidized to form HNCO at higher temperatures. Both HCNO and HNCO can further react with gaseous ethanol to generate EC (Equations (3) and (5)). However, the Gibbs free energy of the reaction between HCNO and ethanol is about 40 kcal/mol, while the Gibbs free energy of the reaction between HNCO and ethanol is about 20 kcal/mol [28]. Thus, the reaction rate between cyanide and gaseous ethanol is much faster than the rate between urea and gaseous ethanol in the formation of EC during the process of distillation.

#### 3.3.2. The Control of EC Formation through the Optimization of the Distillation Device and the Addition of Copper Wires in the Simulated Distillation System

**Figure 8:**
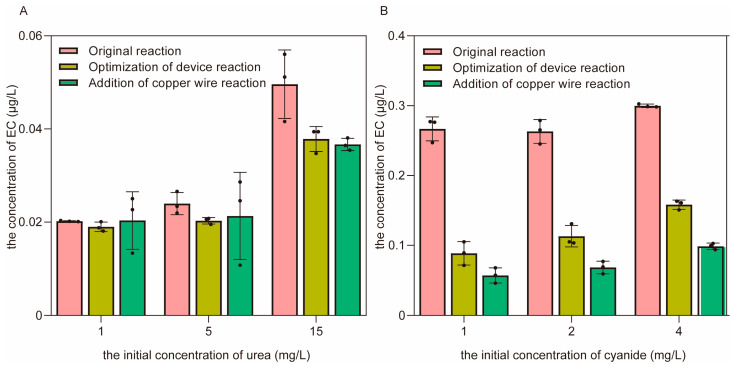
The concentration of EC in the distillate after the optimization of the distillation device and the addition of copper wires. (**A**) The effect of control strategies on the formation of EC using urea as a precursor. (**B**) The effect of control strategies on the formation of EC using cyanide as a precursor.

To control the formation of EC, optimization of the distillation device was performed first. A glass tube that can maintain the temperature at 80 °C (a little higher than the boiling point of ethanol at 78 °C) was extended to decrease the amount of EC brought into the distillate by vapor (Figure 1B) [11,29]. As a result, the formation of EC was significantly reduced by 15.4% when 5.0 mg/L urea was used as the precursor (Figure 8A) and by 57.0% when 2.0 mg/L cyanide was used as the precursor (Figure 8B). The main reason for this result is that the return of vapor can be enhanced when the condensate tube is extended, returning more of the formed EC to the reaction solution.

In addition to the optimization of the distillation device, a copper wire was added as another control strategy for EC (Figure 1C). The results show that the formation of EC using urea as a precursor did not change much (Figure 8A), but the generation of EC using cyanide as a precursor decreased by 37.5% (Figure 8B). The main reason for this phenomenon was that the HCN released from cyanide evaporated with ethanol, and the vapor combined with the copper wire to form a copper–cyanide complex, which further reacted with ethanol to generate EC during distillation, resulting in the formation of EC on the surface of the copper wire [30]. Hence, the concentration of EC in the distillate obviously decreased after adding the copper wire. As for the reaction between urea and ethanol, the orthocyanic acid volatilized by urea could not combine with the copper wire; thus, there was little effect on the formation of EC using urea as a precursor after the addition of the copper wire. In general, the combination of optimizing the distillation device and adding a copper wire was an effective control strategy to reduce the concentration of EC in the simulated distillation system.

### 3.4. The Most Effective Strategy to Reduce the Concentration of EC in Different Flavors of Baijiu

**Figure 9:**
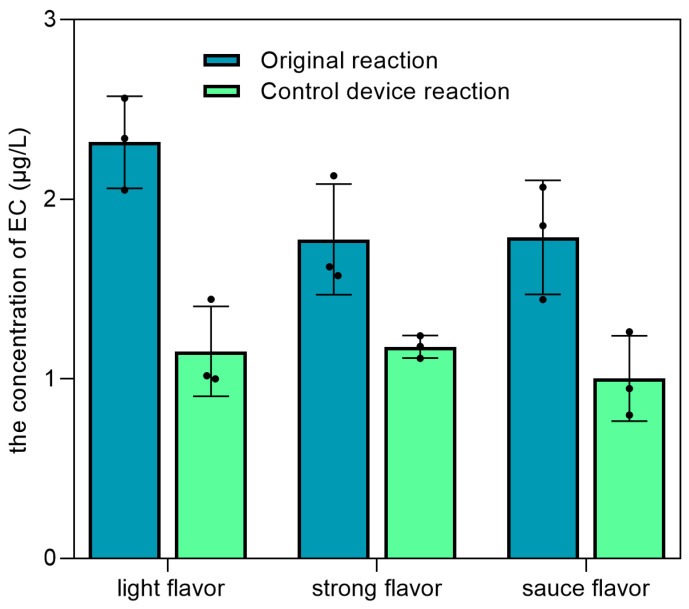
The concentration of EC in distilled spirits after the optimization of distillation for different flavors of Baijiu.

Based on the available strategies for the simulated distillation system, the distillation of fermented grains was performed for different flavors of Baijiu. The results show that the concentration of EC in distilled spirits was reduced by 33.7–50.2% after optimizing the distillation device and adding copper wires (Figure 9), indicating that this method is feasible for controlling the formation of EC in Baijiu. These results are consistent with previous research on the control of EC at relatively low levels through distillation at a higher rate of reflux [9] and on the reaction mechanism between cyanide and copper [31]. However, the catalytic efficiency of copper in controlling the formation of EC was limited for Brazilian sugar cane spirits. Nevertheless, although the concentration of EC was shown to be reduced by 89.91–92.76% through secondary distillation in previous reports, the aroma was severely lost in the final distilled spirits [32]. In the industrial production of Baijiu, a unique distillation process is carried out using a still (a piece of distillation apparatus) [33]. Based on the results of the above research, it is possible to optimize the distillation apparatus by increasing the number of distillation columns and filling them with a large number of copper wires to control the EC content in Baijiu, which has great potential for industrial applications. Thus, the strategy used in this study has obvious advantages in the effective control of EC and the maintenance of Baijiu quality.

## 4. Conclusions

In summary, according to the detection of EC and its precursors in our simulated systems of fermentation, the main precursors of EC are urea and cyanide during the process of brewing for different flavors of Baijiu. However, the dominant stage in which EC formation occurs is during the process of distillation rather than fermentation. In addition, based on simulated aqueous reactions between ethanol and urea or cyanide, the effects of temperature, pH value, alcohol concentration and metal ions on the formation of EC were confirmed. Moreover, through a comparison of reaction rates, the main precursor of EC during the process of distillation was identified as cyanide. Based on the analysis of the formation mechanism, a combination of optimizing the distillation device and adding a copper wire was proposed to control the concentration of EC in Baijiu. Furthermore, the effects of this novel strategy were examined in the gaseous reactions between cyanide (2.0 mg/L) and ethanol (20%), and it was found to reduce the concentration of EC by 74.0%. Finally, the feasibility of this strategy was verified using simulated distillations of fermented grains for different flavors of Baijiu, whereby the formation of EC was reduced by 33.7–50.2%.

## Data Availability

The data presented in this study are available on request from the corresponding author.
